# Twenty Years of *Listeria* in Brazil: Occurrence of *Listeria* Species and *Listeria monocytogenes* Serovars in Food Samples in Brazil between 1990 and 2012

**DOI:** 10.1155/2015/540204

**Published:** 2015-10-11

**Authors:** Deyse Christina Vallim, Cristina Barroso Hofer, Rodrigo de Castro Lisbôa, André Victor Barbosa, Leonardo Alves Rusak, Cristhiane Moura Falavina dos Reis, Ernesto Hofer

**Affiliations:** ^1^Laboratório de Zoonoses Bacterianas, Pavilhão Rocha Lima, Instituto Oswaldo Cruz, Fiocruz, Avenida Brasil 4365, Manguinhos, 21040-360 Rio de Janeiro, RJ, Brazil; ^2^Programa de Pós-Graduação em Doenças Infecciosas e Parasitárias, Faculdade de Medicina, Universidade Federal do Rio de Janeiro, Avenida Carlos Chagas Filho 373, CCS, Bloco K, 2° andar, Sala 49, Cidade Universitária, Ilha do Fundão, 21941-902 Rio de Janeiro, RJ, Brazil

## Abstract

*Listeria* spp. isolated from different food products and collected from 12 Brazilian states were sent to the Laboratory of Bacterial Zoonoses (Oswaldo Cruz Institute, Brazil) for identification. The aims of this study were to characterize these isolates, from 1990 to 2012, by using biochemical, morphological, and serotyping tests, and to analyze the distribution of *L. monocytogenes* serotypes on different food products and geographical locations. Serotyping was performed using polyclonal somatic and flagellar antisera. Of 5953 isolates, 5770 were identified as *Listeria* spp., from which 3429 (59.4%) were *L. innocua*, 2248 (38.9%) were *L. monocytogenes*, and 93 (1.6%) were other *Listeria* spp. *L. innocua* was predominantly isolated from 1990 to 2000, while *L. monocytogenes* was from 2001 to 2012. Regarding the serotype distribution in the foods, serotypes 1/2a and 4b were most common in processed meat and ready-to-eat products, respectively; serotypes 1/2a, 1/2b, and 4b were the most common in nonprocessed meat. The results above confirm the presence of the main serotypes of *L. monocytogenes* in different parts of the food chain from three regions of the country and emphasize the importance of improving the control measures, as tolerance zero policy and microbiological surveillance in Brazil.

## 1. Introduction

The genus* Listeria* includes pathogenic species as* Listeria monocytogenes* and* Listeria ivanovii,* the latter is common in warm-blooded animals, and it is widespread in nature. Its ubiquity is evident by the isolation of this microorganism in fecal specimens from healthy hosts, soil, water, waste, vegetables, and processed silage or food (as well as their factories) [1].

*Listeria monocytogenes* differs from most bacterial food pathogens due its ability to survive harsh environment conditions, grow over a wide temperature range (1–45°C), and survive under wide pH range (4.5–9.6), high salt concentration (10 to 15% NaCl), and very low water activity (aw = 0.94). Therefore it can grow in different types of food products [2].

From a public health perspective*, L. monocytogenes* (mainly the serotypes 1/2a, 1/2b, and 4b) is responsible for severe syndromes in humans, such as meningitis, septicemia, abortion, and febrile enteritis. Its main impact is in immunocompromised individuals, with a high fatality rate of 20% to 30% [3]. As it is a foodborne pathogen, individuals are infected predominantly through contaminated food consumption [4].

Although sporadic cases of listeriosis have been reported in Brazil [5–8], there is no information on foodborne outbreaks involving* L. monocytogenes* [9, 10]. On the other hand, different foods have been recognized as potential sources of this pathogen [6–8, 11–18], but there is no systematic evaluation of these products over time.

In Brazil the Normative Ruling Number 09, promulgated on 8 April 2009 by the Ministry of Agriculture, Cattle Raising and Supply, established criteria and procedures for the implementation of* L. monocytogenes* Control Procedures in ready-to-eat products of animal origin in order to monitor and ensure the safety of these products. These procedures are supervised by a national program called Federal Inspection Service (SIF). The National Agency of Sanitary Surveillance also has the RDC (Board Resolution) Number 12 on 2 January 2001, which establishes the absence of this pathogen in 25 g sample of cheese [19, 20].

Since there is no compulsory notification for cases of Listeriosis in Brazil, Sanitary Surveillance and Public Health has trouble to identify the occurrence of outbreaks, and only isolated cases are reported, which may explain the few listeriosis cases in the literature and the absence of outbreaks reports in our country.

The aims of this study were to (i) identify the species and serotypes of* Listeria* isolated from different food products and regions of Brazil and (ii) analyze the presence of main serotypes of* L. monocytogenes* in different foods and geographical locations of Brazil in an attempt to present a more detailed view of the distribution of* Listeria* spp. in the country.

These isolates were received by the Laboratory of Bacterial Zoonoses (LABZOO) at Oswaldo Cruz Institute (IOC), FIOCRUZ, Rio de Janeiro, Brazil, for identification and serotyping.

## 2. Materials and Methods

### 2.1. Bacterial Cultures

From 1990 to 2012 the laboratory received, from different sources (industries, public universities, research institutes, and other public institutions), 5953 isolates suspected of being* Listeria* spp. for identification, isolated from several food products of 12 different Brazilian states. All these isolates were received in nutrient agar.

### 2.2. Identification of* Listeria* spp

All isolates were screened through hemolysis and colony morphology on Columbia agar containing 5% defibrinated sheep blood, evaluation of the mobility to 25–28°C by stab-inoculation in semisolid agar (tryptose broth containing 0,4% agar), and Gram staining. In this initial screening, 183 (3.07%) isolates were discarded which includes 156 nonmotile Gram positive cocci and 27 motile Gram negative rods. Consequently, 5770 (96.9%) isolates went to further identification and serotyping. The identification was performed using previously published morphological and biochemical tests characterized by catalase, motility, and biochemical tests including acid production from D-xylose, D-mannitol, L-rhamnose, and *α*-methyl-D-mannoside. When necessary,* L. monocytogenes* was differentiated from other species of* Listeria* by using API* Listeria* (BioMeriéux) kit. The final confirmation was provided by Christie-Atkins-Munch-Peterson (CAMP) Test [21].

All isolates identified as* Listeria* were stored in Brain Heart Infusion (Difco) with 20% (v/v) glycerol at −80°C. All the* Listeria* isolates were deposited in the Culture Collection of* Listeria* (CLIST/LABZOO).

### 2.3. *Listeria* spp. Serotyping

The isolates were serotyped using polyclonal antisera produced against* Listeria* somatic and flagellar antigens previously manufactured in the LABZOO according to the method described by Seeliger and Höhne [22].

### 2.4. Statistical Analysis

All the isolates were tabulated using Excel 2007 software. Subsequently, the distribution of all continuous variables, means, medians, and interquartile values were studied. The frequency of all the categorical variables was described. Bivariate analysis of continuous variables was performed using Student's *t*-test or Mann-Whitney test if the variable did not follow normal distribution. Bivariate analysis of categorical variables was performed using Fisher exact test. Statistical analysis was performed using the statistical package STATA version 13.0, Texas, USA.

## 3. Results

The sources of* Listeria* isolates sent to the lab are geographically depicted in Table 1. Of those, 4714 (81.7%) were meat products (processed or/and not processed). Most of the strains (1474, 31.27%) were from poultry products and originated from Goiás, a state in the Middle-West of Brazil.

A total of 1404 (24.3%) strains were isolated from processed meat products (cooked, cured, or smoked products), with the most frequent (900 strains) being mixed sausages (chicken and pork) from two Southeastern Brazilian states. As for dairy products, the home-made cheese from the Southern Brazilian state of Rio Grande do Sul was the main origin.

As can be seen in Table 1, there was a predominance of the southeastern region (states of Minas Gerais, Rio de Janeiro, and São Paulo), with 2711 strains (46.9%), followed by the Midwestern region (states of Mato Grosso and Goiás) with 1817 strains (31.4%), mainly from meat products. These two regions contributed a total of 4528 strains of* Listeria* (78.3%). On a smaller scale, the South and Northeast regions totalized 1078 (18.6%) and 164 strains (2.82%), respectively, mainly from meat and dairy products. Among the states with isolated* Listeria* spp. in dairy products, São Paulo and Rio Grande do Sul contributed with more strains. While São Paulo had a similar number of* Listeria* strains from milk and cheese, in Rio Grande do Sul cheese was the major source (OR = 8.8, 95% CI = 5.2–14.9, *p* < 0.01).

**Figure 1:**
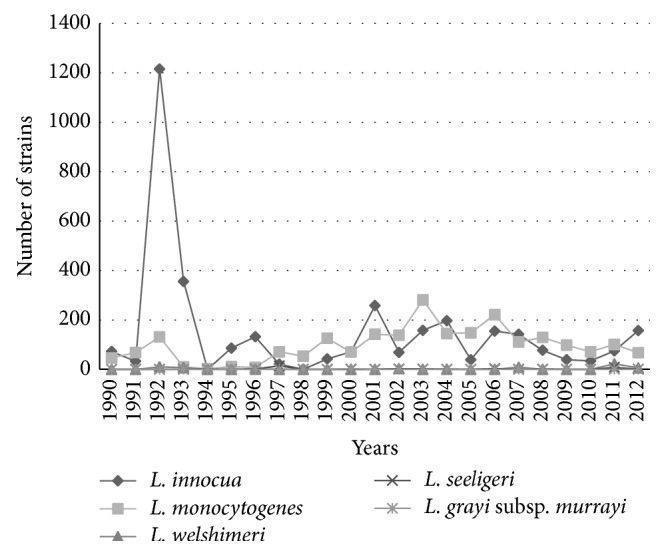
Temporal distribution of* Listeria* spp. isolated from food in Brazil from 1990 to 2012.

The characterization of the species in the period of 1990–2012 (Figure 1) revealed the prevalence of* L. innocua* (3429, 59.4%) and* L. monocytogenes* (2248, 38.9%) and discrete occurrences of* L. welshimeri*,* L. seeligeri,* and* L. grayi *subsp.* murrayi* (93, 1.6%). However,* L. ivanovii* was not isolated from any of the food materials.

The analysis of the trend isolation of* L. monocytogenes* and* L. innocua* during two periods, 1990–2000 and 2001–2012, (number of strains in the period/total strains for the species) showed that* L. innocua* in the first period amounted to 58.9% (2029/3429) and to 40.8% (1400/3429) in the second period. As for* L. monocytogenes*, it amounted to 26.5% (596/2248) in the first period and reached 73.4% (1652/2248) in the second (OR = 3.92, 95% CI = 3.50–4.39, *p* < 0.01).

In species distribution by geographical origin (Table 2), four states, Paraíba, Mato Grosso, São Paulo, and Rio Grande do Sul, had higher levels of* L. monocytogenes* strains (1456 of 2030 strains, 71.7%), surpassing the other* Listeria* spp. isolated, while this was not observed in the other states (792 of 3740 strains, 21.2%) (OR = 9.4, 95% CI = 8.3–10.7, *p* < 0.01). In contrast, in the other states, the prevalence of* L. innocua* (2876 of 3740 strains, 76.9%) was higher (OR = 8.9, 95% CI = 8.9–10.1, *p* < 0.01).

Based on the regional distribution of the species, of the 4692 strains from Northeast, Midwest, and Southeast regions, 2954 (62.9%) were identified as* L. innocua*, while 1684 (35.8%) were identified as* L. monocytogenes*. In the South region, with 1078 strains,* L. monocytogenes* was more frequent (564-52.3%) than* L. innocua* (475-44%) (OR = 2.1, 95% CI = 1.8–2.4, *p* < 0.01), a result biased by the sampling from Rio Grande do Sul.

Regarding the distribution of* L. monocytogenes* serotypes in food products, Table 3 shows that those referred to as the most potentially pathogenic isolates to human consumers, 1/2a, 1/2b, and 4b, were more prominent at 30.3% (1751/5770), rising to 36.8% when serotype 1/2c samples (2129/5770) were included in the calculation. Moreover, the predominance of serotype 4b on most products is evident, except in processed meat, where serotype 1/2a was predominant. When we considered all products, the serotypes 1/2a and 4b presented a similar contribution (10,97% and 11,29%, resp.).

The serotypes 3a, 3b, 3c, 4c, 4e, and 7 and nontypable samples, with rough R-forms, reached a minimum level (119–2.06%), and the isolation of the serotype 4e in milk samples from São Paulo and unprocessed meats (beef and chicken) from Rio Grande do Sul is also noteworthy.

*L. innocua* constituted the largest number of strains (3429 of 5770 strains, 59.4%), of which 2943 were identified as serotype 6a, isolated mainly in unprocessed (poultry) and processed meat (sausages), totaling 2554 of 2943 strains (86,8%).

## 4. Discussion

In the period of 1990–2000, several human listeriosis outbreaks were described in the world with food products as their source; consequently, an intense policy with the implementation of control measures in food industries by Hazard Analysis and Critical Control Points (HACCP) and risk analysis were adopted mainly in Europe and North America. In addition, there are specific recommendations for persons at higher risk for listeriosis. There was also an effort to strengthen quantitative microbiological criteria, with a zero tolerance policy for foods that support and favor the multiplication of bacteria [3, 23]. It should be noted that the policy of zero tolerance has been criticized on the basis that the level of contamination by* L. monocytogenes* in marketed products is often very low and in some developed countries this measure revealed no significant change on listeriosis incidents [24].

In the present study, the laboratory received suspected isolates of* Listeria* spp. However, the sender did not inform if they had established a standard microbiological acceptability of the product or product batches based on it or the presence or number of bacterial masses per unit area or batch. There was also no information about where the products came from, if they were from other geographical areas of Brazil, or if they were imported or would be exported.

The data in Table 1 shows how common members of the genus* Listeria* isolated from foods are in the four regions of Brazil. The lack of samples from the North of Brazil does not indicate that this microorganism is not present in this area. In fact, [25] reported the isolation of* Listeria* spp. in beef in the city of Belém, state of Pará (one of the main cities from Northern Brazil).

Quantitative variations occurred mainly due to the higher concentration of industries that processed food products of animal origin, in particular meat products, mainly from Middle-West, Southeast, and South regions and these are sold to domestic and international markets. On a smaller scale, but present in all regions examined, are dairy products; their marketing is more restricted to the area surrounding the manufacturing site and many products are identified as artisanal.

From an epidemiological point of view, the movement of food carriers is probably an important route for* L. monocytogenes* spread to domestic consumers and those from other regions and/or countries [26].

As for the temporal distribution of isolates characterized into species in the two periods (1990–2000 and 2001–2012) (Figure 1),* L. innocua* had marked predominance in the first period when compared to* L. monocytogenes*. In the second period, the opposite was seen, and we hypothesized that a possible cause for the difference was the improvement of isolation technique by the senders of the samples, as a result of implemented methods for analysis of foods such as the introduction of new selective culture media [27, 28]. Another important detail was that the technical guidance stating that a number of 5 or more suspected isolated colonies in a selective media should be screened for* Listeria* spp. decreased the number of false-positives. In the confirmatory phenotypic analysis, the hemolysis test would frequently yield false-negative results, and this is a critical test in the differentiation between* L. monocytogenes* and* L. innocua.* To overcome this problem, the CAMP test, the alanyl peptidase detection, or molecular typing [28] is recommended.

As for the other species (Figure 1 and Table 2), there was a low incidence of* L. welshimeri, L. seeligeri,* and* L. grayi* subsp.* murrayi* (93 strains, 1.6%) and absence of* L. ivanovii* isolates, according to the findings of Gianfranceschi et al., 2003 [29]. In Table 2 it was observed that four states located in each region revealed the prevalence of* L. monocytogenes*, in opposition to the other areas where* L. innocua* had a higher incidence. Analyzing the results, it can be assumed that there exists a correlation with the type of product, especially meat products (processed and unprocessed) of avian origin, particularly composed of poultry meat sausages. This situation is depicted in isolates from Bahia, Goiás, Minas Gerais, Rio de Janeiro, and Santa Catarina. It was observed that in isolates from unprocessed meat products (cattle, sheep, and pigs) the occurrence of* L. innocua* was not as relevant and in some situations* L. monocytogenes* had a higher incidence in meat products from avian sources in states of Mato Grosso, São Paulo, and Rio Grande do Sul. A hypothesis for the variations in prevalence of the species, whatever the food analyzed is, is that* L. innocua*, when present in equal or greater numbers, tends to overgrow* L. monocytogenes* during the stages of selective enrichment, resulting in a smaller number of* L. monocytogenes* colonies and greater difficulty in viewing them in isolation medium [28].

The presence of* L. monocytogenes* serotypes isolated from various types of food (Table 3) reproduces previous observations from both national [6–8, 11, 16, 17] and international investigations [3, 21, 30] demonstrating the predominant and cosmopolitan nature of the 1/2a, 1/2b, and 4b serotypes, but with increased detection of serotype 1/2c. Overall, these serotypes were found in all products analyzed, with serotype 4b isolates prevailing in ready-to-eat products, serotype 1/2a prevailing in processed meat, and a similar distribution of the serotypes 1/2a, 1/2b, and 4b in nonprocessed meat.

From an epidemiological standpoint, serotyping has a very limited discriminatory level, and only three serotypes (1/2a, 1/2b, and 4b) have a predominant role in human disease processes [31], although presumably serotyping could allow investigators to understand changes in the temporal occurrence of serotypes by geographical region and a particular food type. Nevertheless, serotyping information is necessary to establish the association of serotypes and outbreaks [32].

The observations of Parihar et al. [32] are supported by numerous references about the predominance of serotype 1/2a in clinical cases in Sweden, perhaps as a result of its predominance in many foods, including processed foods. This situation was also reported in other European countries and in the U.S., with serotype 1/2a isolates from food and clinical cases reported more frequently than 4b [23], in gastrointestinal listeriosis [3], or non-outbreak-associated cases of* L. monocytogenes* infection [28]. In our research, the difference between the two serotypes (4b with 652 isolates and 1/2a with 633 isolates) is not significant, even when the isolates related to food without identification are not considered. The frequency of serotypes 1/2b and 1/2c was consistent with previous findings with meat products as the main source of isolation [6, 8, 29]. The antigenic characterization of the predominant species in isolates,* L. innocua*, demonstrated the clear predominance of serotype 6a, mainly in isolates from unprocessed meat products (poultry) and processed meat (sausages containing beef and poultry) as well as in cheeses. The prevalence of other species, identified as* L. welshimeri, L. seeligeri,* and* L. grayi* subsp.* murrayi*, was very low; they were mainly found in nonprocessed meat products.

The association of the resistance of* Listeria* spp. to the conditions imposed by the environment and its wide dissemination in animal sources is a serious problem for the food industry. This situation is a result of the high level of contamination of raw materials originating from animals and vegetables, as these microorganisms may persist for variable periods in the industrial environment. Of particular importance is the ability of* L. monocytogenes* to adhere to surfaces and form biofilm [33]. In the food markets and home environments, a flaw in the cold chain constitutes a risk factor for the growth and dissemination of* Listeria* [3, 34].

The situation depicted by this study demonstrates how easy the transmission of* L. monocytogenes* to the consumer is and the underlying reasons for sporadic cases and outbreaks of listeriosis [3, 35]. Indeed, individuals affected by* Listeria monocytogenes* must encounter the conditions recognized as risk factors [36]. Based on this, attention should be focused on the analysis of risk assessments for ready-to-eat foods, including the establishment of quantitative microbiological criteria as adopted in the U.S. and Europe [3, 35, 37, 38].

Given the results related to ready-to-eat products (Table 3), there is no doubt that consumers are exposed to potential risks of infection, especially the most susceptible population: immune suppressed patients, individuals on extreme age groups, and pregnant women. Furthermore, it is important to take into account the ingested dose of viable bacteria, in addition to the characteristics of the host. This is influenced by the period that the products are kept under refrigeration and the ability of the bacteria to grow in these products [37]. The adoption of a program to reduce the salt concentration in food products (to reduce hypertension) in France raised the hypothesis that this public health measure may have increased the chance of* L. monocytogenes* contamination in foods (mostly meat and fish) and increased the disease incidence [38, 39].

Although the results presented here point to the presence of the main serovars of* L. monocytogenes* in different foods, there are no reports of outbreaks of listeriosis in the country, only a few sporadic cases. This fact can be explained by the absence of mandatory reporting in cases of listeriosis, since there is no compulsory notification for cases of listeriosis in Brazil, and also brings up the possibility that those responsible for the diagnosis do not have proper training to identify this pathogen.

In summary, our work depicts a current overview of the genus* Listeria* distribution in primary foods used as raw material in large national food industry that remain in the final product even after processing.

The eradication of* L. monocytogenes* in foods and processing environments is a difficult task due to the ability of this pathogen to adapt to different harsh conditions. Therefore, it is fundamental the application of HACCP and GMP (Good Manufacturing Practices) programs in industries, a stronger microbiological surveillance and greater dissemination of information about listeriosis (especially for risk groups). Furthermore a network bringing together the public health and food surveillance is necessary in order to gather more efforts to monitor and reduce the risks of food contamination by* L. monocytogenes* in Brazil.

## Figures and Tables

**Table 1:** Geographical distribution of *Listeria* spp. strains by food type in Brazil. 1990–2012.

State	Region^*∗*^	Milk	Cheese	Meat		Ready-to-eat	Vegetables	Unknow source^*∗∗*^	Total	
				Nonprocessed	Processed				*N*	%
Alagoas	NE		34						34	0.58
Bahia	NE	4	1	39	19	2			65	1.12
Paraíba	NE	25	8						33	0.57
Pernambuco	NE		28	2	2				32	0.55
Goiás	MW			1474	60				1534	26.58
Mato Grosso	MW		17	43	223				283	4.9
Minas Gerais	SE		87	346	472		17		922	15.97
Rio de Janeiro	SE	2	36	474	57	8	26		603	10.45
São Paulo	SE	92	95	352	428	114	56	49	1186	20.55
Paraná	S		12	29	4				45	0.77
Santa Catarina	S		42	380	4	79			505	8.75
Rio Grande do Sul	S	22	200	171	135				528	9.15

Total		145	560	3310	1404	203	99	49	5770	

^*∗*^NE, Northeast; MW, Middle-West; SE, Southeast; S, South.

^*∗∗*^Food source not informed by the senders.

**Table 2:** Geographical distribution of *Listeria* spp. isolates, in Brazil. 1990–2012.

States	Species					Total	
	*L. innocua *	*L. monocytogenes *	*L. welshimeri *	*L. seeligeri *	*L. grayi *subsp.* murrayi *	*N*	%
Alagoas	22	12				34	0.58
Bahia	57	6			2	65	1.12
Paraíba	6	27				33	0.57
Pernambuco	28	4				32	0.55
Goiás	1350	169	15			1534	26.58
Mato Grosso	64	216		3		283	4.9
Minas Gerais	763	154	3	2		922	15.97
Rio de Janeiro	330	259		14		603	10.45
São Paulo	334	837	13	2		1186	20.55
Paraná	28	14	3			45	0.77
Santa Catarina	298	174	25	8		505	8.75
Rio Grande do Sul	149	376		3		528	9.15

Total							
*N*	3429	2248	59	32	2	5770	99.8
%	59.42	38.96	1.02	0.55	0.03		

**Table 3:** Species and serovar frequency of *Listeria* spp. isolates from food, in Brazil. Period 1990–2012.

Food type	*L. innocua *			*L. monocytogenes *												*L. welshimeri *	*L. seeligeri *	*L. grayi *	Total	
	6a	6b	NT^*∗*^	1/2a	1/2b	1/2c	3a	3b	3c	4b	4c	4e	7	NT^*∗*^	R^*∗∗*^				*N*	%
*Meat*:																				
Nonprocessed	1939	90	233	244	250	140	6	20	2	275		28	1	2	4	51	25		3310	57.37
Processed	615	53	27	239	113	144	5	8		163	2	4		18		8	5		1404	24.33
Ready-to-eat	387	38	45	139	90	80		1		195		13	1	4			2	2	997	17.28
Unknown	2			11	13	14				19									59	1.02

Total																				
*N*	2943	181	305	633	466	378	11	29	2	652	2	45	2	24	4	59	32	2	5770	100
%	51.0	3.14	5.29	10.97	8.08	6.55	0.19	0.51	0.04	11.29	0.04	0.78	0.04	0.41	0.06	1.02	0.55	0.04		

^*∗*^NT = nontypable (smooth form).

^*∗∗*^R = rough form.
